# Lockdown Duration and Training Intensity Affect Sleep Behavior in an International Sample of 1,454 Elite Athletes

**DOI:** 10.3389/fphys.2022.904778

**Published:** 2022-06-15

**Authors:** Mohamed Romdhani, Hugh H. K. Fullagar, Jacopo A. Vitale, Mathieu Nédélec, Dale E. Rae, Achraf Ammar, Hamdi Chtourou, Ramzi A. Al Horani, Helmi Ben Saad, Nicola Luigi Bragazzi, Gürhan Dönmez, Ismail Dergaa, Tarak Driss, Abdulaziz Farooq, Omar Hammouda, Nesrine Harroum, Bahar Hassanmirzaei, Karim Khalladi, Syrine Khemila, Leonardo Jose Mataruna-Dos-Santos, Imen Moussa-Chamari, Iñigo Mujika, Hussein Muñoz Helú, Amin Norouzi Fashkhami, Laisa Liane Paineiras-Domingos, Mehrshad Rahbari Khaneghah, Yoshitomo Saita, Nizar Souissi, Khaled Trabelsi, Jad Adrian Washif, Johanna Weber, Piotr Zmijewski, Lee Taylor, Sergio Garbarino, Karim Chamari

**Affiliations:** ^1^ High Institute of Sport and Physical Education, Sfax University, Sfax, Tunisia; ^2^ Physical Activity, Sport and Health, UR18JS01, National Observatory of Sports, Tunis, Tunisia; ^3^ School of Sport, Exercise and Rehabilitation, Faculty of Health, University of Technology Sydney, Sydney, NSW, Australia; ^4^ IRCCS Istituto Ortopedico Galeazzi, Milan, Italy; ^5^ Research Unit, Laboratory Sport, Expertise and Performance (EA7370), The French National Institute of Sport (INSEP), Paris, France; ^6^ Division of Exercise Science and Sports Medicine, Department of Human Biology, Faculty of Health Sciences, University of Cape Town, Cape Town, South Africa; ^7^ Institute of Sport Science, Otto-Von-Guericke University, Magdeburg, Germany; ^8^ Interdisciplinary Laboratory in Neurosciences, Physiology and Psychology: Physical Activity, Health and Learning (LINP2), UFR STAPS, UPL, Paris Nanterre University, Nanterre, France; ^9^ Department of Exercise Science, Yarmouk University, Irbid, Jordan; ^10^ Laboratoire de Recherche (LR12SP09) “Insuffisance Cardiaque”, Hôpital Farhat HACHED, Faculté de Médecine de Sousse, Université de Sousse, Sousse, Tunisia; ^11^ Department of Health Sciences, Postgraduate School of Public Health, University of Genoa, Genoa, Italy; ^12^ Laboratory for Industrial and Applied Mathematics, Department of Mathematics and Statistics, York University, Toronto, ON, Canada; ^13^ Department of Sports Medicine, Hacettepe University School of Medicine, Ankara, Turkey; ^14^ Primary Health Care Corporation (PHCC), Doha, Qatar; ^15^ Aspetar, Orthopaedic and Sports Medicine Hospital, FIFA Medical Center of Excellence, Doha, Qatar; ^16^ Research Laboratory, Molecular Bases of Human Pathology, LR19ES13, Faculty of Medicine, University of Sfax, Sfax, Tunisia; ^17^ School of Kinesiology and Physical Activity Science, Faculty of Medicine, Montreal University, Montreal, QC, Canada; ^18^ Sports Medicine Research Center, Neuroscience Institute, Tehran University of Medical Sciences, Tehran, Iran; ^19^ Iran Football Medical Assessment and Rehabilitation Center, IFMARC, FIFA Medical Center of Excellence, Tehran, Iran; ^20^ High Institute of Sports and Physical Education Ksar-Said, Manouba University, Manouba, Tunisia; ^21^ Department of Sport Management, Faculty of Management, Canadian University of Dubai, Dubai, United Arab Emirates; ^22^ Coventry University—Centre for Trust, Peace and Social Relation, Coventry, United Kingdom, United Kingdom; ^23^ Physical Education Department, College of Education, Qatar University, Doha, Qatar; ^24^ Department of Physiology, Faculty of Medicine and Nursing, University of the Basque Country, Bilbao, Spain; ^25^ Exercise Science Laboratory, School of Kinesiology, Faculty of Medicine, Universidad Finis Terrae, Santiago, Chile; ^26^ Department of Economic-Administrative Sciences, Universidad Autónoma de Occidente, Culiacán, Mexico; ^27^ Esteghlal Physiotherapy Clinic, EPC, Teheran, Iran; ^28^ Programa de Pós-Graduação em Ciências Médicas, Universidade do Estado do Rio de Janeiro, Rio de Janeiro, Brazil; ^29^ Departamento de Fisioterapia, Instituto Multidisciplinar de Reabilitação e Saúde, Universidade Federal da Bahia, Salvador, Brazil; ^30^ Department of Sports and Regenerative Medicine, Juntendo University, Tokyo, Japan; ^31^ Research Laboratory: Education, Motricity, Sport and Health, EM2S, LR19JS01, University of Sfax, Sfax, Tunisia; ^32^ Sports Performance Division, National Sports Institute of Malaysia, Kuala Lumpur, Malaysia; ^33^ Neurocognition and Action—Biomechanics, Bielefeld University, Bielefeld, Germany; ^34^ Institute for Sports Science, Christian-Albrechts-University of Kiel, Kiel, Germany; ^35^ Józef Piłsudski University of Physical Education in Warsaw, Warsaw, Poland; ^36^ School of Sport, Exercise and Health Sciences, National Center for Sport and Exercise Medicine (NCSEM), Loughborough University, Loughborough, United Kingdom; ^37^ Sport and Exercise Discipline Group, Faculty of Health, University of Technology Sydney (UTS), Sydney, NSW, Australia; ^38^ Department of Neuroscience, Rehabilitation, Ophthalmology, Genetics and Maternal-Infantile Sciences, University of Genoa, Genoa, Italy; ^39^ Post-Graduate School of Occupational Medicine, Università Cattolica del Sacro Cuore, Roma, Italy

**Keywords:** highly-trained athletes, home-confinement duration, pandemic (COVID-19), training load, sleep disturbance

## Abstract

**Objective:** To investigate the effect of 1) lockdown duration and 2) training intensity on sleep quality and insomnia symptoms in elite athletes.

**Methods:** 1,454 elite athletes (24.1 ± 6.7 years; 42% female; 41% individual sports) from 40 countries answered a retrospective, cross-sectional, web-based questionnaire relating to their behavioral habits pre- and during- COVID-19 lockdown, including: 1) Pittsburgh sleep quality index (PSQI); 2) Insomnia severity index (ISI); bespoke questions about 3) napping; and 4) training behaviors. The association between dependent (PSQI and ISI) and independent variables (sleep, napping and training behaviors) was determined with multiple regression and is reported as semi-partial correlation coefficient squared (in percentage).

**Results:** 15% of the sample spent < 1 month, 27% spent 1–2 months and 58% spent > 2 months in lockdown. 29% self-reported maintaining the same training intensity during-lockdown whilst 71% reduced training intensity. PSQI (4.1 ± 2.4 to 5.8 ± 3.1; mean difference (MD): 1.7; 95% confidence interval of the difference (95% CI): 1.6–1.9) and ISI (5.1 ± 4.7 to 7.7 ± 6.4; MD: 2.6; 95% CI: 2.3–2.9) scores were higher during-compared to pre-lockdown, associated (all *p* < 0.001) with longer sleep onset latency (PSQI: 28%; ISI: 23%), later bedtime (PSQI: 13%; ISI: 14%) and later preferred time of day to train (PSQI: 9%; ISI: 5%) during-lockdown. Those who reduced training intensity during-lockdown showed higher PSQI (*p* < 0.001; MD: 1.25; 95% CI: 0.87–1.63) and ISI (*p* < 0.001; MD: 2.5; 95% CI: 1.72–3.27) scores compared to those who maintained training intensity. Although PSQI score was not affected by the lockdown duration, ISI score was higher in athletes who spent > 2 months confined compared to those who spent < 1 month (*p* < 0.001; MD: 1.28; 95% CI: 0.26–2.3).

**Conclusion:** Reducing training intensity during the COVID-19-induced lockdown was associated with lower sleep quality and higher insomnia severity in elite athletes. Lockdown duration had further disrupting effects on elite athletes’ sleep behavior. These findings could be of relevance in future lockdown or lockdown-like situations (e.g., prolonged illness, injury, and quarantine after international travel).

## Introduction

To mitigate the spread of the COVID-19 virus, governments implemented restrictive mandates including; social distancing, to stay at home, curfews and quarantines (termed “lockdown” from here on). Elite athletes stopped or drastically reduced their training, competition and travel due to lockdowns. This increased sedentary behaviors, altered nutritional behaviors and reduced motivation (Pillay et al., 2020; Romdhani et al., 2022). Lifestyle factors, including social interaction, sleep patterns, physical activity and mental health were also affected in this population (Pillay et al., 2020; Romdhani et al., 2022; Facer-Childs et al., 2021; Vitale et al., 2021).

Regarding the reciprocal interaction between sleep and athletic training, better sleep quality and quantity are associated with regular physical activity (Chennaoui et al., 2015). Indeed, training and competition are potential *zeitgebers* (from German; time givers) for human circadian rhythms, including those of elite athlete (Davenne, 2009; Vitale et al., 2019). Furthermore, it has been reported that changes in training loads (i.e., volume and intensity) may interfere with subsequent sleep architecture (Youngstedt, 2005; Davenne, 2009; Chennaoui et al., 2015). Lockdown aside, elite athletes are known for their lower sleep quality compared to the general population, due to sport-related factors (e.g., long haul travel, intensive training, media commitment), resulting in high prevalence of insomnia, greater sleep fragmentation and non-restorative sleep (Gupta et al., 2016; Roberts et al., 2019). Lockdown reduced these sport related factors’ impact on athletes. Thus, an improvement in elite athletes’ sleep quality during-lockdown was expected. However, the myriad lockdown-mediated disruptions (Pillay et al., 2020; Romdhani et al., 2022; Facer-Childs et al., 2021; Vitale et al., 2021) negatively affected elite athletes’ sleep and circadian rhythms more severely than that seen in the general population and non-elite athletes (Romdhani et al., 2022).

Appropriate sleep (duration and quality) supports the maintenance of physical and psychological health in athletes (Nedelec et al., 2018; Walsh et al., 2021). Indeed, inadequate sleep quality and quantity are associated with increased injury risk (Milewski et al., 2014), reduced physical and cognitive performance (Romdhani et al., 2019; 2021a) and compromised recovery (Rae et al., 2017; Romdhani et al., 2019). Additionally, the lockdown-induced reduction in training load (volume and/or intensity) could lead to detraining (Mujika and Padilla, 2000a, b). Detraining is defined as the partial or complete loss of training-induced adaptations subsequent to an insufficient training stimulus (Mujika and Padilla, 2000b). According to Mujika and Padilla (2000a, b), the duration of the detraining process [i.e., long term (more than 4 weeks) or short term (less than 4 weeks)] can impact the return to sport after lockdown eases. The fact that lockdown duration was not the same across the world (ranging from two to several weeks) could affect athletes from various countries differentially. To the best of the authors’ knowledge, only two studies have discussed the effect of lockdown duration on sleep behavior in the general population and reported conflicting results (Salfi et al., 2020; Wang et al., 2020). However, little is known about the effects of lockdown duration on elite athletes’ sleep quality and insomnia symptoms. Additionally, how training intensity during-lockdown potentially affected elite athletes’ sleep behavior has not been explored.

Therefore, it appears important to determine the effect of COVID-19-induced lockdown on elite athletes’ sleep quality and insomnia severity. Further, understanding the duration of lockdown and/or how this moderated training intensity and their subsequent singular or combined effects on sleep quality and insomnia severity in elite athletes seems prudent. For our study, it was hypothesized that in elite athletes: 1) sleep and training behaviors will be affected by the lockdown; 2) athletes who maintained the same training intensity during - compared to pre-lockdown will be less affected by sleep disruption than those who reduced training intensity; and 3) the duration of lockdown will negatively affect sleep quality.

## Population and Methods

The present study is part of a bigger project investigating the effect of COVID-19 lockdown on sleep and circadian rhythms in an international sample of 3911 athletes (from different backgrounds). Part of this project has been published elsewhere (Romdhani et al., 2022) (providing full and open-access descriptions of methods and data), comparing the effect of lockdown on athletes’ sleep behavior based on sub-groups (elite vs. non-elite; male vs. female; individual vs. team; and old vs. young). The present data focuses on elite athletes only (*n* = 1,454; male = 57%), with greater depth of data analysis and more nuanced and expanded discussion thereof, compared to the previous publication (Romdhani et al., 2022).

### Participants and Reliability

Eligibility criteria for participants were: 1) ≥18 years of age; 2) classified as an elite athlete (individual or team sport) of both sexes; and 3) had been under conditions of lockdown for at least 2 weeks. Elite athletes are defined as the highest level of competitiveness whether having full income from sport or not (Swann et al., 2015). Please see supplementary file 1 (Part I; question 6) for further understanding.

### Procedure

Informed consent was provided by participants under ethical approval from an institutional review board, in the spirit of the Helsinki Declaration. Data were processed anonymously and according to the guidelines of the “General Data Protection Regulation” (gdpr-info.eu).

A retrospective, international, cross-sectional, web-based questionnaire was developed by thirty-five multidisciplinary scientists and academics, from forty research centers. It surveyed: 1) the Pittsburgh sleep quality index (PSQI); 2) Insomnia severity index (ISI); bespoke questions about 3) napping; and 4) training behaviors in elite athletes who had experienced a period of lockdown, with comparisons made between “during-lockdown” and the “month preceding lockdown” (i.e., baseline). The original English survey was translated into nine languages (Arabic, French, Italian, Japanese, Malay, Persian, Portuguese, Spanish and Turkish). The survey questions underwent translation and back-translation, performed by the research team (including at least one native speaker and a topic expert). Thereafter, 1% of the participants (42 athletes) responded to these new questions twice (1 week apart), assessing reliability [ranging from good (*r* = 0.83) to very good (r = 0.97)]. The survey was launched online through social media (e.g., Facebook, Twitter, WhatsApp and e-mail), opening 8 July 2020 and closing 30 September 2020.

### Survey

The survey had five sections and is provided as Supplementary Material.

Section 1: Explanation of the study; invited volunteers to confirm eligibility; provide consent to participate; and encouraged respondents to respond as accurately as possible.

Section 2: Demographic and lockdown questions.

Athletes were asked about their sex, age, country of residence, lockdown duration (i.e., less than 1 month, between 1 and 2 months and over 2 months), and sport discipline (team/individual). Participants were also asked about their level of competition (amateur, sub-elite and elite) and only elite athletes’ data are discussed here (Swann et al., 2015).

Section 3: Questions related to training.

Athletes were asked about their preferred time of day (TOD) to train (hh:mm, regardless of the number of training sessions per day) and the number of training sessions per week (training frequency). Further, they were subjectively asked whether or not (yes, no or I do not know) they were maintaining the same training intensity (unlike volume/frequency of training) during-lockdown compared to pre-lockdown.

Section 4: Pittsburgh Sleep Quality Index (PSQI) and Insomnia Severity Index (ISI).

The PSQI assessed subjective sleep quality pre- and during-lockdown. PSQI score ranges from 0–21, with a score of ≥5 indicating a poor sleep quality and a score of ≥8 indicating very poor sleep quality (Buysse et al., 1989). The PSQI questionnaire also determined self-reported bed, wake and mid-sleep times (hh:mm), total sleep time (TST, min), time in bed (TIB, min), total sleep time per day (TST + nap duration, min), sleep onset latency (SOL, min) and sleep efficiency (SE, %; TST/TIB × 100).

The ISI contains seven questions to assess severity of insomnia symptoms. Total ISI score ranges from 0–28, indicative of moderate (15–21) or severe (22–28) clinical insomnia (Bastien et al., 2001).

Section 5: Napping behavior.

Athletes were also asked about their napping behavior [i.e., timing (preferred TOD, hh:mm), duration (min) and frequency (number per week)] through a set of customized, bespoke questions.

### Statistical Analyses

Statistical analysis was performed using SPSS (IBM Corp. IBM SPSS Statistics for Windows, Version 26. Armonk, NY, United States: IBM Corp) and figures were created using GraphPad (GraphPad Software, San Diego, CA, United States). Differences in variables between pre- and during-lockdown were analyzed using a *t* test for dependent samples. The interaction between lockdown and training intensity (pre/during * yes/no) and the lockdown duration (pre/during * ≤1 month/1–2 months/≥ 2 months) were analyzed using a mixed design analysis of variance (ANOVA). Significant effects underwent a Bonferroni post-hoc test with Cohen’s (*d*) effect size subsequently calculated, qualitatively interpreted as small (*d* < 0.5), moderate (0.5 ≤ *d* < 0.8) and large (*d ≥* 0.8) (Cohen, 1992). The multiple regression model was based on delta variation (Δ%; the % of change of each variable from pre-to during-lockdown) according to the formula [100 * (during-lockdown—pre-lockdown)/pre-lockdown], as per (Romdhani et al., 2022). After checking for the assumptions (e.g., correlation between independent and dependent variables and colinearity), all the independent variables were entered into the model using a stepwise method. We report the *R* square (*R* ^2^, the proportion of variance in the dependent variable explained by the independent variables) for the entire model as well as the semi-partial correlation coefficient squared (sr^2^) to indicate the unique contribution (in percentage) of each independent variable within the model. All values within the text are reported as mean ± standard deviation (SD). However, data in figures are presented as 95% of the confidence interval. Alpha was set at *p* < 0.05.

## Results

### Participants

The global sample (*n* = 1,454; 24.1 ± 6.7 years) included athletes from 40 countries, continentally located as: Asia (61%; *n* = 884); Europe (17%; *n* = 243); North and South America (13%; *n* = 190); Africa (9%; *n* = 133); and Australia (0.2%; *n* = 4). Athletes were from team (59%; *n* = 852) and individual (41%; *n* = 602) sports, with 57% (*n* = 828) male and 42% (*n* = 613) female [1% (*n* = 13) preferring not to declare their sex]. 29% (*n* = 351) reported maintaining the same intensity of training during-compared to pre-lockdown, 71% (*n* = 1,025) reduced training intensity and 5% (*n* = 78) were not sure and they were, therefore, removed from the binary “training intensity” comparison. 222 athletes (15%) spent less than 1 month in lockdown, compared to 389 (27%) who spent between 1 and 2 months and 843 (58%) who spent more than 2 months in lockdown.

### The Effects of Lockdown

**FIGURE 1:**
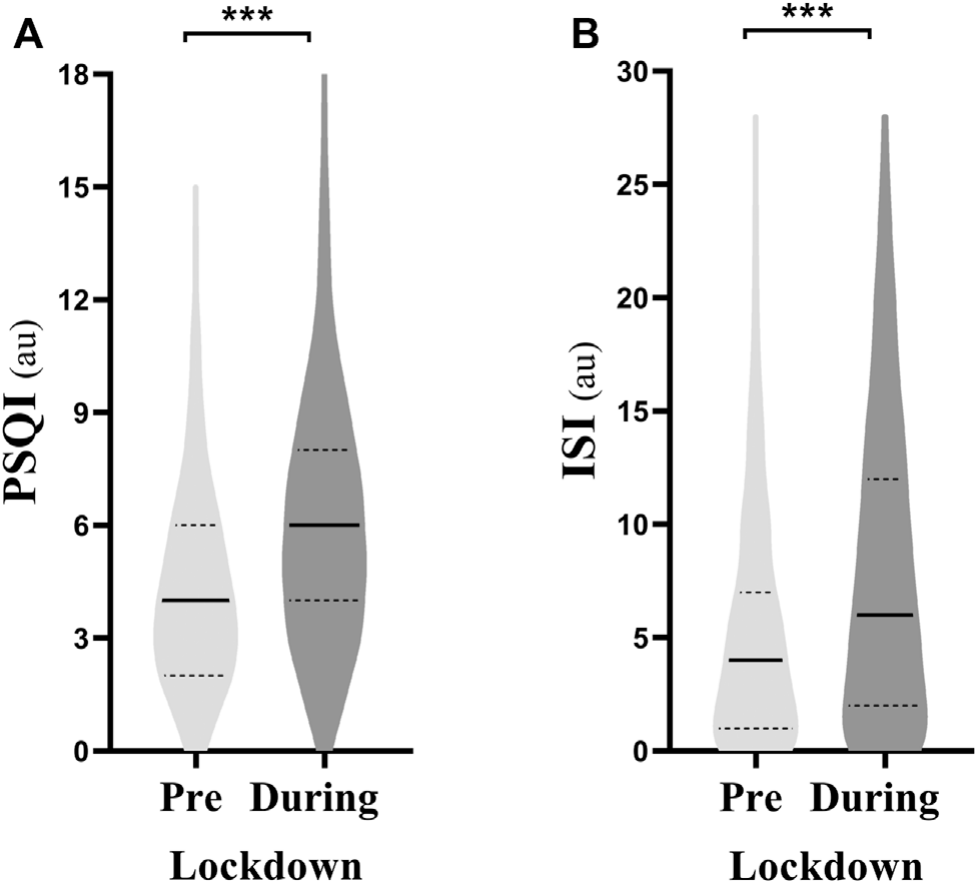
Violin plot of **(A)** Pittsburgh sleep quality index (PSQI) and **(B)** insomnia severity index (ISI) pre- and during-lockdown. *** means significant within subject effect of the lockdown at *p* < 0.001. Significance is assessed by a paired sample *t* test, au, arbitrary unit.

PSQI (4.1 ± 2.4 to 5.8 ± 3.1) and ISI (5.1 ± 4.7 to 7.7 ± 6.4) scores were higher during-compared to pre-lockdown (Figure 1). Pre-lockdown, 38.5% of the athletes reported poor sleep quality and 9% reported very poor sleep quality. These proportions increased to 64% (poor sleep quality) and 28.2% (very poor sleep quality) during-lockdown. Further, 4.4% and 0.6% reported moderate and severe insomnia pre-lockdown, which increased to 13 and 3.3% during-lockdown, respectively. The effect of lockdown on the overall sample is presented in Table 1.

**TABLE 1:** Statistical parameters relating to changes in sleep and training behaviors in response to lockdown.

Variable	Lockdown		*t*	*N* =	*p* <	*d*	MD	95% CI
	Pre	During						
PSQI score (au)	4.1 ± 2.4	5.8 ± 3.1	22.7	1,455	0.001	0.61	1.7	1.6 to 1.9
ISI score (au)	5 ± 4.7	7.6 ± 6.4	18.2	1,455	0.001	0.46	2.6	2.3 to 2.9
Bedtime (hh:mm)	23:14 ± 1:10	00:41 ± 1:51	32.1	1,455	0.001	0.87	1:27	1:22 to 1:32
Wake-up time (hh:mm)	7:17 ± 1:30	9:55 ± 2:32	42.7	1,455	0.001	1.27	2:38	2:31 to 2:45
Mid-sleep time (hh:mm)	3:15 ± 1:06	5:17 ± 2:01	42.1	1,455	0.001	1.24	2:02	1:56 to 2:08
Total sleep time (min)	455 ± 67	501 ± 86	19.8	1,455	0.001	0.59	45	41 to 50
Time in Bed (min)	503 ± 76	591 ± 102	33.3	1,455	0.001	0.97	87	82 to 92
Sleep Efficiency (%)	90.3 ± 9.1	85.1 ± 9.8	18.6	1,455	0.001	0.54	−5.1	−4.6 to −5.7
Sleep Onset Latency (min)	19.6 ± 13.8	35.1 ± 27.4	24.6	1,455	0.001	0.71	15.5	14.2 to 16.7
Nap Duration (min)	14.7 ± 20.6	21.1 ± 25.2	8.8	1,455	0.001	0.29	6.3	4.9 to 7.8
Nap Timing (hh:mm)	14:15 ± 1:29	14:34 ± 1:33	4.55	983	0.001	0.18	0:19	0:12 to 0:26
24 h sleep duration (min)	470 ± 70	522 ± 90	21.3	1,455	0.001	0.64	52	47 to 56
Preferred training TOD (hh:mm)	13:54 ± 4:31	14:32 ± 4:32	4.55	1,455	0.001	0.11	0:38	0:24 to 0:52
Training sessions (N°·week^−1^)	3.7 ± 2.4	5.6 ± 2.3	26.7	1,455	0.001	0.81	−1.8	−1.7 to −1.9

Dependent t-tests were used to compare variables measured pre and during-lockdown. 95% CI: 95% Confidence interval; au, arbitrary unit; *d*, Cohen’s effect size; h, hour; ISI, insomnia severity index; MD, mean difference; min, minutes; N°, number; PSQI, pittsburgh sleep quality index; TOD, time of day.

### The Effects of Training Intensity

**FIGURE 2:**
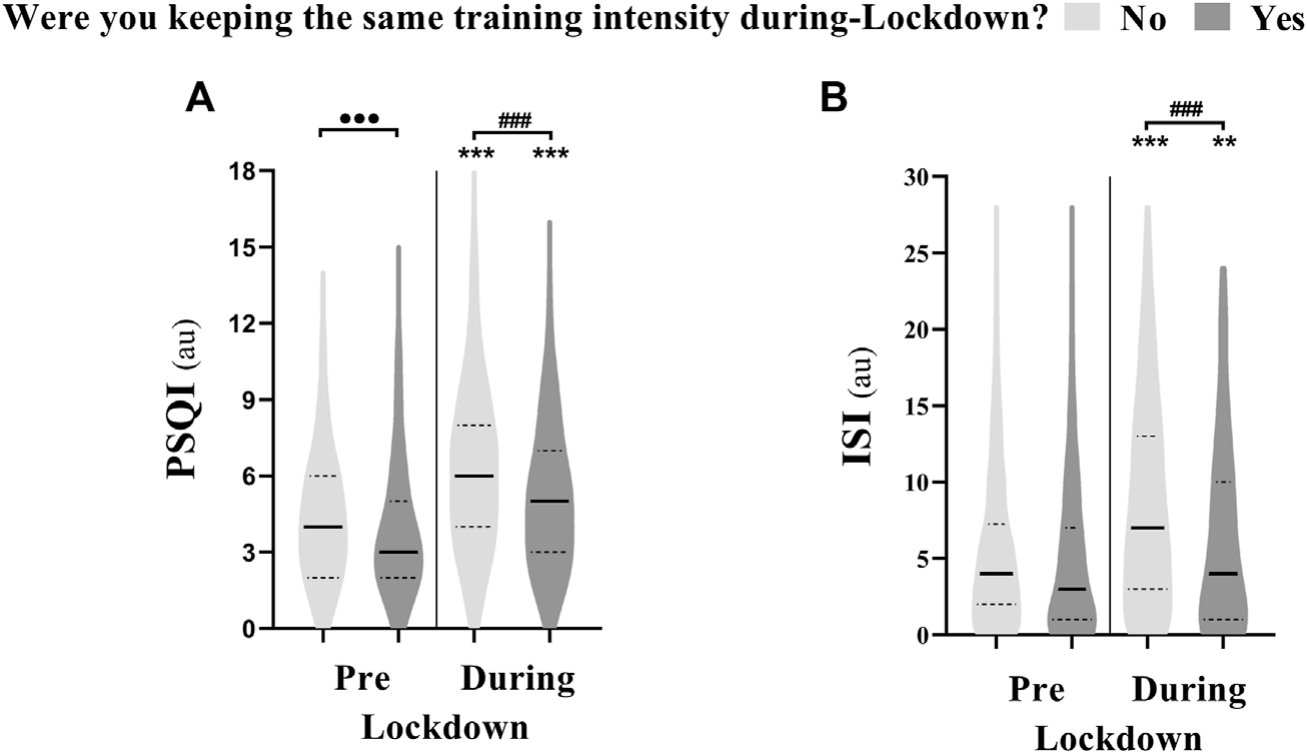
Violin plot showing the difference in **(A)** Pittsburgh sleep quality index (PSQI) and **(B)** insomnia severity index (ISI) between athletes who were keeping the same training intensity during-compared to pre-lockdown (*n* = 351; Yes) and those who were not (*n* = 1,025; No). *** means significant within subject effect of the lockdown at *p* < 0.001. ⋅⋅⋅ means significant between subject effect pre-lockdown at *p* < 0.001. **###** means significant between subject effect during-lockdown at *p* < 0.001. Significance is assessed by a mixed design ANOVA and the Bonferroni post-hoc test.

The effect of training intensity during-lockdown on sleep parameters is presented in Figure 2. Athletes who kept the same training intensity during-lockdown showed a smaller increase in PSQI and ISI compared to those who reduced training intensity. 75% of athletes who reported poor sleep quality and 80% of athletes who reported moderate insomnia during-lockdown also reported that they reduced training intensity compared to pre-lockdown. Further, 80% of the athletes reporting very poor sleep quality and 87% of athletes that reported severe insomnia during-lockdown reduced training intensity during-lockdown. The effect of training intensity on training and napping behavior is presented in Table 2.

**TABLE 2:** Training and napping behavior pre- and during-lockdown according to training intensity.

	Training Intensity			
	Pre-lockdown		During-lockdown	
	Yes	No	Yes	No
Training sessions (N°·week^−1^)	5.7 ± 2.5	5.6 ± 2.2	4.4 ± 2.5^a^	3.6 ± 2.3^a^ ^,^ ^b^
Preferred training TOD (hh:mm)	14:16 ± 4:38	13:56 ± 4:31	14:33 ± 4:37	14:41 ± 4:31^a^
Nap Duration (min)	12.7 ± 19.3	15.5 ± 20.9	17.5 ± 22.3^a^	22.2 ± 25.8^a^ ^,^ ^b^
Nap Timing (hh:mm)	14:25 ± 1:36	14:16 ± 1:31	14:29 ± 1:35	14:42 ± 1:33^a^ ^,^ ^b^

Mixed ANOVA with repeated measure and the Bonferroni post-hoc test were used to compare the within subject effect of lockdown (pre-compared to during-lockdown), and the between subject; the effect of training intensity.

a Means significant within subject effect at p < 0.05.

b Means a significant between subject effect at p < 0.05.

h, hour; min, minutes; N°, number; TOD, time of day.

### The Effects of Lockdown Duration

**FIGURE 3:**
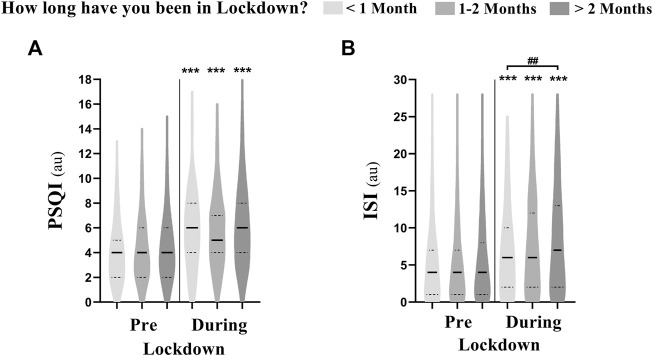
Violin plot showing the difference in **(A)** Pittsburgh sleep quality index (PSQI) and **(B)** insomnia severity index (ISI) between athletes who were in lockdown for <1 month (*n* = 222), 1–2 months (*n* = 389) and >2 months (*n* = 843). *** means significant within subject effect of the lockdown at *p* < 0.001. ### means significant between subject effect during-lockdown at *p* < 0.001. Significance is assessed by a mixed design ANOVA and the Bonferroni post-hoc test.

The effect of lockdown duration on sleep parameters is presented in Figure 3. PSQI scores increased from pre-to during-lockdown independently of lockdown duration. However, ISI scores were higher during-lockdown in athletes who spent more than 2 months (8.1 ± 6.6) compared to those who spent less than 1 month (6.7 ± 5.7) under lockdown. In addition, 64% of the athletes reporting moderate insomnia during-lockdown were in lockdown for over than 2 months (compared to 23% for 1–2 months and 12% for less than 1 month). Likewise, 73% of the athletes reporting severe insomnia during-lockdown spent more than 2 months in lockdown (compared to 21% for 1–2 months and 6% for less than 1 month). The effect of lockdown duration on training and napping behavior is presented in Table 3.

**TABLE 3:** Training and napping behavior pre- and during-lockdown according to the lockdown duration.

	Lockdown duration					
	Pre-lockdown			During-lockdown		
	<1 month	1–2 months	>2 months	<1 month	1–2 months	>2 months
Training sessions (N°·week^−1^)	5.5 ± 2.3	5.5 ± 2.2	5.6 ± 2.3	3.4 ± 2.3^a^	3.7 ± 2.3^a^	3.9 ± 2.4^a^ ^,^ ^b^
Preferred training TOD (hh:mm)	14:00 ± 4:30	13:40 ± 4:28	14:10 ± 4:34	14:10 ± 4:34	14:47 ± 4:22^a^	14:42 ± 4:37^a^
Nap Duration (min)	12.6 ± 19.1	14.2 ± 19.5	15.6 ± 20.6	19.1 ± 25.1^a^	20.4 ± 24.3^a^	22.1 ± 25.3^a^
Nap Timing (hh:mm)	14:21 ± 1:38	14:17 ± 1:26	14:17 ± 1:34	14:40 ± 1:42	14:36 ± 1:33^a^	14:38 ± 1:34^a^

Mixed ANOVA with repeated measure and the Bonferroni post-hoc test were used to compare the within subject effect of lockdown (pre-compared to during-lockdown), and the between subject; the effect of the lockdown duration.

a Means significant within subject effect at p < 0.05

b Means a significant between subject effect at p < 0.05.

h, hour; min, minutes; N°, number; TOD, time of day.

### Multiple Regression

The multiple regression model for PSQI (F_(6, 1448)_ = 76.37; *p* < 0.001; *R* ^2^ = 0.24) and ISI (F_(6, 1448)_ = 24.68; *p* < 0.001; *R* ^2^ = 0.09) were both significant. The change in PSQI score from pre-to during-lockdown was positively associated with the change of SOL (*t* = 12.1; *p* < 0.001; 28%), bedtime (*t* = 5.7; *p* < 0.001; 13%), training TOD (*t* = 3.9; *p* < 0.001; 9%), nap duration (*t* = 2.1; *p* < 0.001; 5%), and negatively associated with SE (*t* = −11.1; *p* < 0.001; −26%) and TST (*t* = −6.1; *p* < 0.001; −14%). Moreover, the change in ISI score from pre-to during-lockdown was positively associated with the change of SOL (*t* = 9.1; *p* < 0.001; 23%), bedtime (*t* = 5.5; *p* < 0.001; 14%), nap duration (*t* = 3.1; *p* < 0.001; 8%), training TOD (*t* = 2.2; *p* < 0.001; 5%), and negatively associated with training intensity (*t* = -2.9; *p* < 0.001; −8%).

## Discussion

Lockdown reduced sleep quality and increased insomnia in elite athletes, primarily associated with longer sleep onset latency (SOL) and later bedtime, preferred time of day (TOD) to train, and daytime napping. Further, elite athletes increased their total sleep time (TST) and the time in bed (TIB) during-lockdown. Those who maintained the same training intensity reported lower insomnia and better sleep quality during-lockdown compared to elite athletes that reduced training intensity. The duration of lockdown had detrimental effects on athletes’ sleep-wake behavior and training, with long lockdown periods being associated with higher insomnia severity and poorer sleep quality.

### The Effects of Lockdown

Lockdown reduced sleep quality and increased insomnia severity in the current sample of elite athletes. Two out of three athletes reported poor, and one out of four reported very poor sleep quality during-lockdown. Furthermore, the proportion of athletes reporting moderate insomnia increased three-fold and severe insomnia six-fold, during-lockdown. Overall, all measured sleep metrics were negatively affected by the lockdown. For example, comparing pre-to during-lockdown, alongside increases in PSQI, increases in SOL, later bedtime and later preferred TOD to train were also observed. Although speculative, several lockdown-induced behavioral changes may explain this evidenced disruption to sleep and training (Pillay et al., 2020; Romdhani et al., 2022; Facer-Childs et al., 2021). Indeed, longer SOL has been shown to be secondary to a higher caffeine and alcohol consumption, longer and later daytime naps, and increased nighttime feeding behavior (Romdhani et al., 2022). In addition, elite and sub-elite athletes reported higher depression, anxiety and stress symptoms during-lockdown, which can negatively affect sleep quality (Facer-Childs et al., 2021). Also, elite athletes have been shown to increase the time spent in front of screens (e.g., TV, video and mobile games) during-lockdown, which can reduce sleep quality and lengthen SOL (Pillay et al., 2020; Facer-Childs et al., 2021).

Interestingly, ∼40% of elite athletes in our study reported poor sleep quality pre-lockdown, in line with previous studies (Gupta et al., 2016; Roberts et al., 2019; Romdhani et al., 2022; Walsh et al., 2021). Therefore, to find an increase in sleep duration, time in bed and the frequency and duration of daytime naps during-lockdown was not surprising. It was suggested that the increased TST and TIB during-lockdown are secondary to a chronic sleep restriction experienced by elite athletes pre-lockdown (Facer-Childs et al., 2021). Of interest, this extra sleep duration was associated with a better sleep quality during-lockdown (*p* < 0.001; 14%). This suggests that elite athletes were compensating for lower sleep quality during-lockdown, by a longer TST (+45 min) and TIB (+87 min). Also, during-lockdown, later bedtimes (+87 min) were associated with lower sleep quality (*p* < 0.001; 13%) and higher insomnia (*p* < 0.001; 14%). Indeed, the shift toward eveningness (misplaced sleep timing) during-lockdown, despite the longer TST and TIB, reduced sleep quality. In line with our findings, delayed bedtime was related to poorer sleep quality regardless of age, sex and sleep duration (Merikanto et al., 2012), which raises the importance of sleep timing during the day, with delayed bedtimes resulting in decreased sleep quality. In addition, the shift toward eveningness was mediated by later preferred TOD to train during-lockdown (Romdhani et al., 2022). Training sessions (especially team training) and competition are a potent *zeitgeber* for elite athletes’ circadian rhythms (Davenne, 2009). With the reduced number of training sessions during-lockdown (Romdhani et al., 2022; Washif et al., 2021), it was not surprising to find disrupted sleep parameters in this sample of elite athletes.

### The Effects of Training Intensity

**FIGURE 4:**
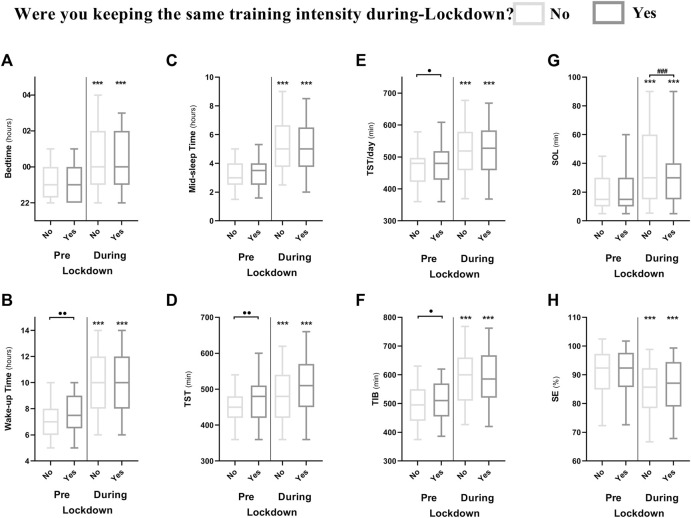
Difference between athletes who were keeping the same training intensity during-compared to pre-lockdown (*n* = 351; Yes) and those who were not (*n* = 1,025; No) in different sleep metrics; **(A)** Bedtime; **(B)** wake time; **(C)** mid-sleep time; **(D)** total sleep time; **(E)** total sleep time per 24 h; **(F)** time in bed; **(G)** sleep onset latency and **(H)** sleep efficiency. *, **and *** means significant within subject effect of the lockdown at *p* < 0.05, *p* < 0.01 and *p* < 0.001, respectively.** ⋅**, **⋅⋅** and **⋅⋅⋅** means significant between subject effect pre-lockdown at *p* < 0.05, *p* < 0.01 and *p* < 0.001, respectively. #, ## and ### means significant between subject effect during-lockdown at *p* < 0.05, *p* < 0.01 and *p* < 0.001, respectively. Significance is assessed by a mixed design ANOVA and the Bonferroni post-hoc test.

Participants were subjectively asked (yes or no question) if they were keeping the same intensity of training during-compared to pre-lockdown. 64% (931 out of 1,454 elite athletes) of the current sample reported poor sleep quality during-lockdown, of whom, 698 (75%) self-reported that they reduced training intensity during-lockdown. Of interest, those who reduced training intensity during-lockdown showed higher insomnia and lower sleep quality. They also reported longer SOL (+5.2 min) and later (+12 min) and longer (+3.4 min) daytime naps. Maintaining training intensity during-lockdown (to values approximate to pre-lockdown) was protective against an unfavorable change in ISI score (*p* < 0.001; −8%) and thus exacerbation of insomnia symptoms. Athletes who maintained training intensity also preserved the same duration and timing of daytime naps, preferred TOD of training sessions and displayed a smaller increase in mid-sleep time; indicative of resisting negative effects of lockdown on training and sleep disruption (Figure 4). Speculatively, maintaining training intensity during-lockdown could have retained sleep drive at a high level and masked the lockdown-induced disruption (e.g., longer SOL, lower sleep efficiency and later bedtime) (Youngstedt, 2005; Davenne, 2009). This indicates that preserving the same training intensity—at least subjectively—compared to pre-lockdown mitigated (at least in part) the lockdown-induced deterioration of sleep quality and insomnia symptoms.

Those who maintained the same training intensity during-lockdown reported better sleep quality, later wake time (+21 min) and longer TST (+10 min) and TIB (+12 min) pre-lockdown compared to those who did not (Figure 4). A possible explanation is that those who maintained training intensity had less circadian interference from potential external changes in scheduling. Indeed, disruptions to normal behavioral schedules (e.g., team training being one of the circadian modulators for elite athletes) can affect sleep quantity and quality (Davenne, 2009). Comparatively, those maintaining a consistent physical activity and sleep routine are more likely to sleep better. Another contributor for those maintaining the same training intensity could have been the assistance from a coach and/or training staff as reported elsewhere (Pillay et al., 2020). However, elite athletes participating in the current study were not asked whether or not they were assisted by training staff, which could limit the potential association of our findings.

### The Effects of Lockdown Duration

**FIGURE 5:**
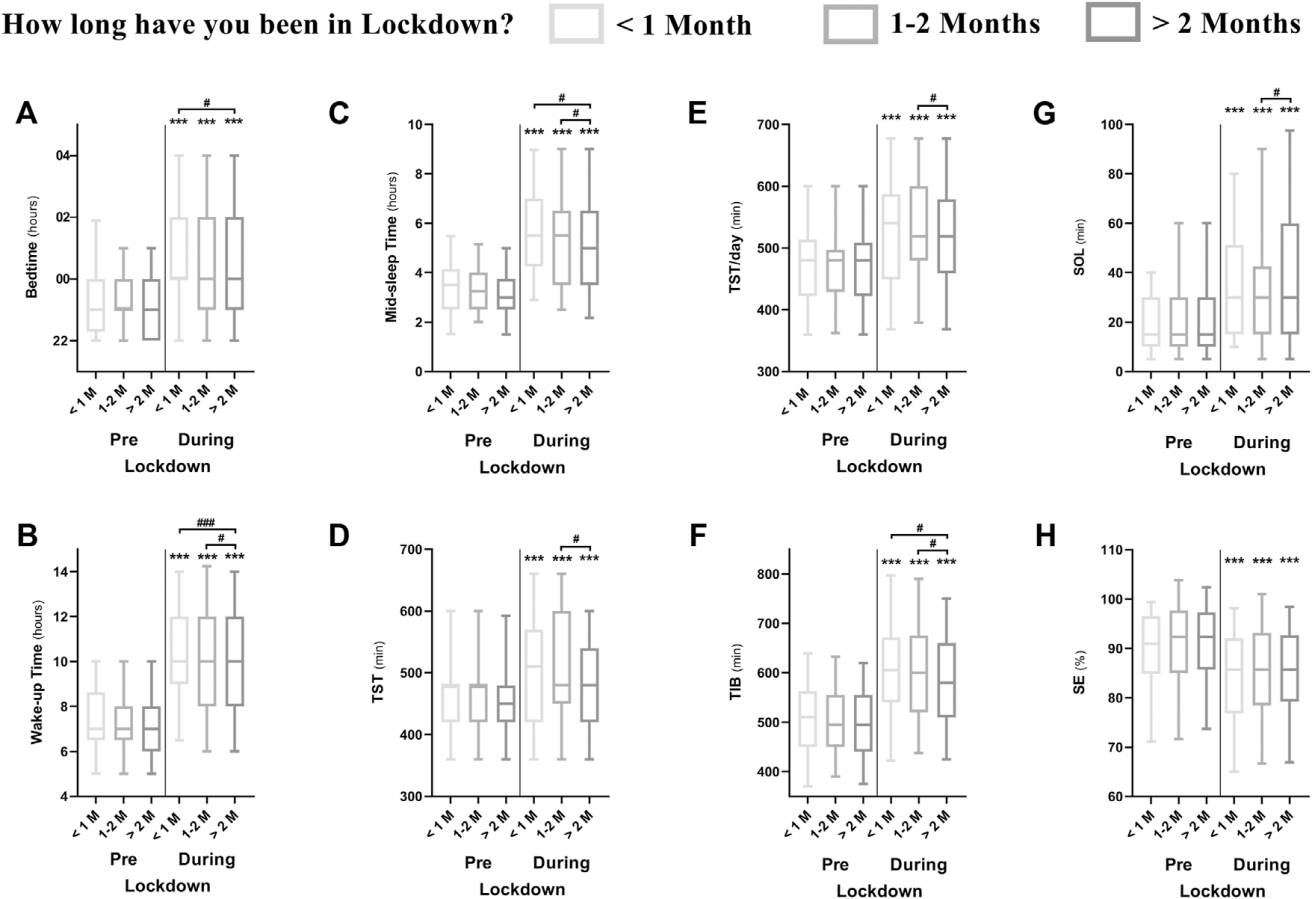
Difference between athletes who were in lockdown for <1 month (*n* = 222), 1–2 months (*n* = 389) and >2 months (*n* = 843) during-compared to pre-lockdown in different sleep metrics; **(A)** Bedtime; **(B)** wake time; **(C)** mid-sleep time; **(D)** total sleep time; **(E)** total sleep time per 24 h; **(F)** time in bed; **(G)** sleep onset latency and **(H)** sleep efficiency. *, **and *** means significant within subject effect of the lockdown at *p* < 0.05, *p* < 0.01 and *p* < 0.001, respectively. #, ## and ### means significant between subject effect during-lockdown at *p* < 0.05, *p* < 0.01 and *p* < 0.001, respectively. Significance is assessed by a mixed design ANOVA and the Bonferroni post-hoc test.

Elite athletes who were locked-down for more than 2 months showed higher ISI scores compared to less than 1 month. Further, they were sleeping and waking earlier, having longer SOL and spending less TIB compared to others (Figure 5). Also, those who were locked-down for more than 1 month (1–2 months and more than 2 months) displayed later preferred TOD to train compared to pre-lockdown, which was associated with lower sleep quality and higher insomnia. These findings indicate that longer lockdown durations could have further disturbing effects on sleep quality and insomnia severity than shorter ones.

Those who spent less than 1 month in lockdown had the lowest number of training sessions per week, with the number of training sessions increasing in those who spent more than 2 months. A straightforward explanation could be that recovery mesocycles were advanced to “make the best” of lockdown restrictions (especially if lockdown followed a period of competition congestion), yet as lockdown continued (e.g., 2 months or more) solutions were required (and found) to resume appropriate training. Additionally, speculation regarding lockdown easing alongside some competition recommencing may have provided impetus for athletes to return to training (in turn leading athletes to sleep and wake earlier).

Those who spent less than 1 month in lockdown showed the strongest shift in bed, wake and mid-sleep times, indicative of higher sleep routine disruption. A possible explanation is that athletes were experiencing a mismatch between circadian and social clocks pre-lockdown, and once the opportunity was available, they shifted to the preferred schedule. Also, those in the first month of lockdown showed the longest TIB. This may indicate that athletes were potentially sleep restricted pre-lockdown, and once the opportunity of extra sleep became available, they took advantage of it to repay sleep debt (Nédélec et al., 2015). Indeed, as aforementioned, elite athletic cohorts have been reported to sleep less than recommended (Sargent et al., 2021; Walsh et al., 2021).

### Strengths and Limitations

To the best of the authors’ knowledge, the current study is the largest study investigating sleep behaviors within the context of lockdown in elite athletes. The survey was developed by an international research team, which ensures that the outcomes of this study will reach a high number of elite athletes, support staff and sport clinicians. The main limitation for the present study is that we had to collect data about the pre-lockdown period by retrospective self-report, which could be subjected to recall bias. The current study was advertised online and it could be subjected to sampling bias (e.g., non-elite claiming that they are elite athletes and/or the concept of an elite athlete in different world regions, and/or not having a precise perception about their actual training level (Swann et al., 2015). Further, the PSQI and ISI were used beyond their original purposes and were adapted to better suit the current study objective. Indeed, whether or not, how, and to which extent this has an effect on the current assumptions is unclear. Although the present sample showed a shift toward eveningness, the effect of lockdown on circadian rhythms within a context of chronotype was not reported here (Rae et al., 2015; Vitale et al., 2015). Athletes were asked to subjectively report whether or not they kept the same training intensity, differently from volume and/or frequency, during-compared to pre-lockdown. Therefore, it is unclear, from the current paradigm, if changing exercise modality and/or other aspects of training could have affected the current conclusions. Also, the lockdown level (e.g., partial or total lockdown) was not the same across the world at the time of survey, which implicate that the current results should be treated with caution. Finally, the potential effect of the environment (e.g., house surface, number of family members, sharing the bed and/or bedroom) on subjective sleep quality was beyond the focus of this study and could be underestimated.

### Practical Applications

Athletes extended their sleep duration and delayed bed and wake time within the first month of the lockdown, indicative of a potential accumulated sleep restriction pre-lockdown and/or a mismatch between biological and social clocks. The preferred time of day to train saw a high (a standard deviation of 4 h 32 min) inter-individual difference pre- and during-lockdown. Therefore, the unique training time dictated by the coach/staff is not always in agreement with the preferences of athletes, especially in team sports. It could be of importance to take individual preferences when planning training sessions and to allow sufficient sleep time to optimize recovery and mental health in elite athletes. However, maintaining training intensity could be key during lockdown-like situations to retain/promote physical and mental health whilst preventing detraining in elite athletes (Mujika and Padilla, 2000a, b).

## Conclusion

This international survey showed that, when compared to pre-COVID-19 pandemic, the early 2020 lockdown resulted in lower sleep quality and higher insomnia symptoms in elite athletes. The latter results were mainly due to longer sleep onset latency and later bedtime. Athletes who were able and/or willing to maintain the same training intensity during-lockdown were less affected by the lockdown-induced sleep disruption. Moreover, the lockdown duration had further disrupting effects on elite athletes’ sleep and insomnia severity. These findings could be of relevance in countries which are still implementing lockdowns, in future lockdown or lockdown-like situations, and/or eventual prophylactic self-isolation after international travel.

## Data Availability

The raw data supporting the conclusion of this article will be made available by the authors, without undue reservation.
